# Impact of epidemiological characteristics of supratentorial gliomas in adults brought about by the 2016 world health organization classification of tumors of the central nervous system

**DOI:** 10.18632/oncotarget.13555

**Published:** 2016-11-24

**Authors:** Haihui Jiang, Yong Cui, Junmei Wang, Song Lin

**Affiliations:** ^1^ Department of Neurosurgery, First Hospital of Tsinghua University, Beijing, China; ^2^ Department of Neurosurgery, Beijing Tiantan Hospital, Capital Medical University, Beijing, China; ^3^ China National Clinical Research Center for Neurological Diseases, Beijing, China; ^4^ Beijing Institute for Brain Disorders and Beijing Key Laboratory of Brain Tumor, Beijing, China; ^5^ Department of Pathology, Beijing Neurosurgical Institute, Beijing, China

**Keywords:** gliomas, classification, epidemiology, impact

## Abstract

The latest World Health Organization (WHO) classification of tumors of the central nervous system (CNS) integrates both histological and molecular features in the definition of diagnostic entities. This new approach enrolls novel entities of gliomas. In this study, we aimed to reveal the epidemiological characteristics, including age at diagnosis, gender ratio, tumor distribution and survival, of these new entities. We retrospectively reclassified 1210 glioma samples according to the 2016 CNS WHO diagnostic criteria. In our cohort, glioblastoma multiforme (GBM) with wildtype isocitrate dehydrogenase (IDH) was the most common malignant tumor in the brain. Almost all gliomas were more prevalent in males, especially in the cluster of WHO grade III gliomas and IDH-wildtype GBM. Age at diagnosis was directly proportional to tumor grade. With respect to the distribution by histology, we found that gliomas concurrent with IDH-mutant and 1p/19q-codeleted or with single IDH-mutant were mainly distributed in frontal lobe, while those with IDH-wildtype were dominant in temporal lobe. Lesions located in insular lobe were more likely to be IDH-mutant astrocytoma. In summary, our results elucidated the epidemiological characteristics as well as the regional constituents of these new gliomas entities, which could bring insights into tumorigenesis and personalized treatment of Chinese glioma population.

## INTRODUCTION

The 2016 World Health Organization (WHO) classification of tumors of the central nervous system (CNS) has successfully updated in May, which promoted the pathology into a molecular era as the result of integration of relevant biomarkers [1]. The 2016 CNS WHO remarkably redefined the diffuse gliomas based on the molecular parameters and traditional histology features. It has not only added newly recognized neoplasms, but also deleted some entities that no longer have diagnostic and/or biological relevance. Most notably, the oligoastrocytoma, a diagnostic category suffered from high interobserver discordance in traditional pathology definition [2–4], could been pertinently reclassified into astrocytoma or oligodendroglioma according to the IDH and 1p/19q status. All these indicated that the component and proportion of diffuse gliomas has virtually changed.

Considering the nonnegligible impact on component and proportion of glioma brought about by the 2016 CNS WHO, more comprehensive re-understanding of the epidemiological characteristics of “new” gliomas in adults appears to be particularly important. At the same time, more studies and clinic works should be carried on to investigate the reliability and validity of the new pathologic classification scheme.

Therefore, in the present study, we retrospectively reviewed 1210 adult patients with supratentorial gliomas in our institution and then reclassified their samples according to the 2016 CNS WHO. In addition, the epidemiological characteristics of these new pathology entities, including age at diagnosis, gender ratio, location and survival time were analyzed.

## RESULTS

### Incidence of the newly diagnosed entities

**Figure 1:**
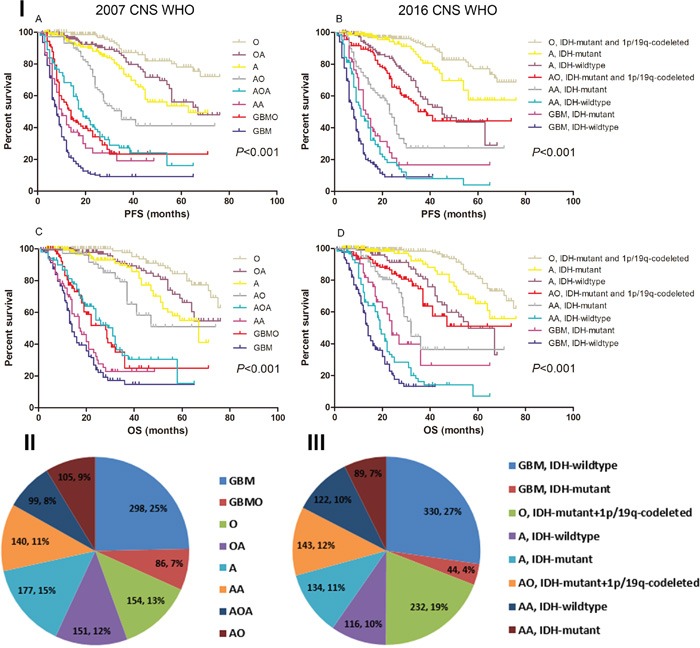
The survival rate and specific distribution of 2007/2016 CNS WHO pathology entities **I**. Kaplan-Meier estimates of survival time in 2007/2016 CNS WHO pathology entities. Both the 2007 and 2016 CNS WHO could categorized patients into eight entities with different survival. **II**. Distribution of diagnostic entities in the 2007 CNS WHO (N=1210). GBM was the most frequently reported malignant tumor in the brain, which accounted for 25.0% of all the glioma. **III**. Distribution of diagnostic entities in the 2016 CNS WHO (N=1210). GBM with IDH-wildtype was the most common malignant tumor in the brain, which accounted for 27.0% of all the glioma.

In our dataset, the most common histologic type in adults with supratentorial lesion was IDH-wildtype GBM (27.0%) (Figure 1). The incidence of grade II, III and IV gliomas was 40.0%, 29.0% and 31.0%, respectively (Supplementary Figure S1). Meanwhile, the most prevalent molecular subtype was gliomas with IDH-wildtype (47.0%), followed by IDH-mutant and 1p/19q-codelted (31.0%), and trailed by IDH-mutant (22.0%) according to the 2016 classification scheme (Supplementary Figure S2). Comparing with 2007 CNS WHO, the oligoastrocytoma was no longer served as an independent diagnostic entity and thereby reclassified into either astrocytoma or oligodendroglioma in the light of 1p/19q and IDH detection results.

Notably, we found the incidence of GBM decreased. According to the 2007 CNS WHO criteria, there were 384 (32.0%) GBM_2007_ [including 298 GBM and 86 glioblastomas with oligodendroglioma component (GBMO)] in our cohort. After the reclassification based on 2016 CNS WHO, only 374 (31.0%) patients were diagnosed with GBM_2016_, including 44 IDH-mutant GBM and 330 IDH-wildtype GBM. The rest of 10 patients who harbored 1p/19q-codeleted and IDH-mutant were reclassified into AO (WHO grade III) (Figure 1 and Supplementary Figure S1). Nevertheless, GBM was still the most frequently reported malignant tumor in the brain, which accounted for 31.0% of all the glioma (Figure 1).

### Relative survival of the newly diagnosed entities

Overall, The higher grade, the shorter survival time (*P*<0.001) (Supplementary Figure S1). The patients concurrent with IDH-mutant and 1p/19q-codelted had the most favorable prognosis, while those with IDH-wildtype showed the shortest survival (*P*<0.001) (Supplementary Figure S2). Interestingly, the Log-Rank analysis revealed that the IDH-mutant GBM showed a tendency of increased survival rate than IDH-wildtype AA, but the difference was not statistically significant [13.0 months *vs*. 11.0 months, *P*=0.146 for progression-free survival (PFS); 24.0 months *vs*. 19.0 months, *P*=0.059 for overall survival (OS)] (Figure 1). Furthermore, we have found that the median PFS and OS of GBM_2016_ were relatively shorter compared with GBM_2007_ (8.0 months *vs*. 9.0 months and 14.0 months *vs*. 15.0 months) (Supplementary Figure S1).

### Distribution of gender and age of the newly diagnosed entities

Almost all gliomas were more prevalent in males, although the gender difference were quite small in the case of WHO grade II gliomas (Table 1). The gender ratio was significantly higher in grade III gliomas compared with grade II or IV gliomas (*P*=0.008, by Chi-Square) (Table 1). Besides, the incidence for male in IDH-wildtype GBM was 1.9 times higher than those in IDH-mutant GBM (*P*=0.05, by Chi-Square) (Table 1).

**Table 1:** Clinical characteristics of different glioma entities

Variables	Number of cases	Median age at diagnosis (years)	Gender ratio (M/F)	Median PFS (months)	Median OS (months)
O, 1p/19q-codeleted and IDH-mutant	232	42	1.2:1	N/A	N/A
A, IDH-mutant	134	38	1.3:1	N/A	N/A
A, IDH-wildtype	116	40.5	1.4:1	45.0	56.0
AO, 1p/19q-codeleted and IDH-mutant	143	42	2.5:1	38.5	N/A
AA, IDH-mutant	89	43	1.9:1	23.0	32.0
AA, IDH-wildtype	122	42	1.8:1	11.0	19.0
GBM, IDH-mutant	44	48	1:1	13.0	24.0
GBM, IDH-wildtype	330	50	1.9:1	8.0	14.0

Abbreviation: PFS, progression-free survival; OS, overall survival; M, male; F, female; IDH, isocitrate dehydrogenase; O, oligodendroglioma; A, astrocytoma; AO, anaplastic oligodendroglioma; AA, anaplastic astrocytoma; GBM, glioblastoma multiforme; N/A, not available.

As for the age distribution, GBM with IDH-wildtype was associated with the highest median age at diagnosis (median age=50 years old). The further subgroup comparison showed that the age at diagnosis was directly proportional to tumor grade (*P*=0.022, by one-way ANOVA) (Table 1).

### Incidence by site and histology of the newly diagnosed entities

**Figure 2:**
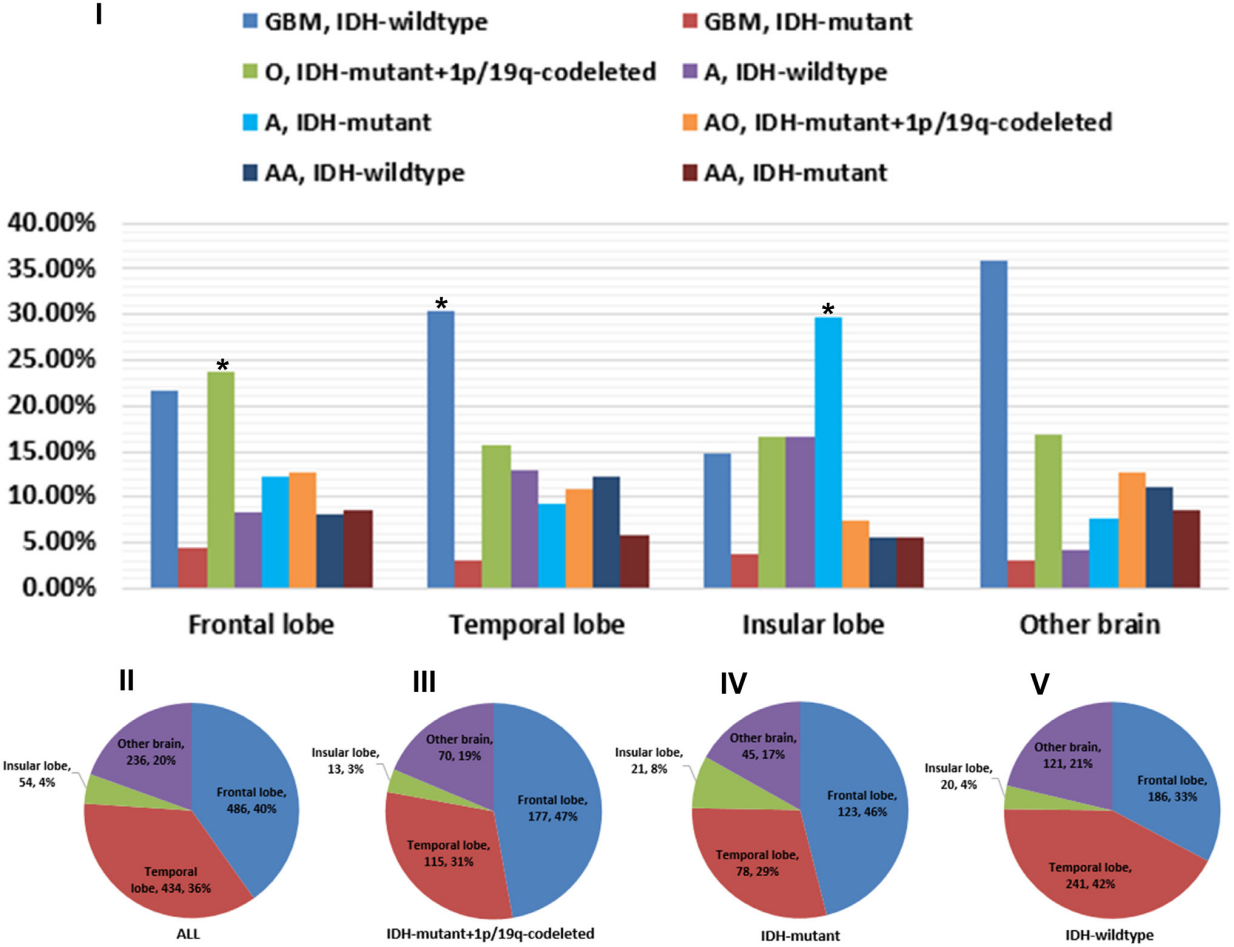
Regional constituents of pathological entities and preferential distribution of different tumor subtypes **I**. Composing proportion of pathological entities in the frontal, temporal, insular and other brain sites. The predominant proportion in frontal, temporal and insular lobe was IDH-mutant and 1p/19q-codeleted O, IDH-wildtype GBM and IDH-mutant A, respectively. **II-V**. Almost 76.0% gliomas occurred in frontal and temporal lobe. Patients concurrent with IDH-mutant and 1p/19q-codeleted or with single IDH-mutant were mainly distributed in frontal lobe, while those with IDH-wildtype were dominant in temporal lobe.

The distribution of gliomas by site was shown in Figure 2. Patients concurrent with IDH-mutant and 1p/19q-codeleted (47.0%) or with single IDH-mutant (46.0%) were mainly distributed in frontal lobe, while those with IDH-wildtype (42.0%) were dominant in temporal lobe (all *P*<0.05, by Chi-Square). The following subgroup analysis confirmed these findings in both astrocytic and oligodendroglial tumors.

Overall, frontal lobe accounted for 40.0%, temporal 36.0%, insular 4.0%, and other brain 20.0% of all gliomas (Figure 2). The percentage of IDH-mutant and 1p/19q-codeleted oligodendroglioma (O) in frontal lobe was 23.7%, which was significantly higher than those of other pathologic types (*P*=0.001, by Chi-Square). Similarly, the proportion of pathologic type in temporal lobe was driven by IDH-wildtype GBM (30.4%), even though the P value was marginally significant (*P*=0.066, by Chi-Square). In insular lobe, the most common pathologic type was IDH-mutant astrocytoma (A) (29.6%) (*P*<0.001, by Chi-Square).

### Validation of the prognostic potential of the 2016 WHO classification system

Univariate analysis demonstrated that age 45 ≥years, gross total resection (GTR), karnofsky performance score (KPS) ≥70, 1p/19q-codeleted, IDH-mutant, O^6^-methylguanine-DNA methyltransfer (MGMT) promoter Methylation and 2016 CNS WHO were associated with prognosis (*P*<0.05, data not shown). The 2016 CNS WHO categorized the whole cohort into eight subtypes with significantly distinct survival (*P*<0.001) (Figure 1). In the further Cox proportional hazard model enrolled all these parameters, 2016 CNS WHO was confirmed as an independent factor affecting PFS and OS. The hazard ratio (HR) of the 2016 CNS WHO was 0.932 [95% confidence interval (CI), 0.897-0.967; *P*<0.001] for PFS and 0.919 (95% CI, 0.880-0.961; *P*<0.001) for OS (Table 2).

**Table 2:** Multivariate analysis to identify factors that predict survival in all patients

Variables	Hazard Ratio (HR)	95% Confidence Interval (CI)	P value
Factors associated with PFS			
Age ≥45	1.333	1.129-1.574	0.001
2016 WHO CNS	0.932	0.897-0.967	<0.001
1p/19q-codeleted	0.432	0.323–0.578	<0.001
IDH-mutant	0.403	0.317-0.512	<0.001
MGMT-Methylated	0.748	0.616-0.907	0.003
GTR	0.789	0.659-0.945	0.010
Factors associated with OS			
Age ≥45	1.269	1.047-1.538	0.015
2016 WHO CNS	0.919	0.880-0.961	<0.001
1p/19q-codeleted	0.421	0.295–0.602	<0.001
IDH-mutant	0.354	0.265-0.473	<0.001
MGMT-Methylated	0.793	0.634-0.990	0.041
GTR	0.694	0.565-0.853	0.001

Abbreviation: HR, hazard ratio; 95% CI, 95% confidence interval; PFS, progression-free survival; OS, overall survival; WHO, world health organization; CNS, central nervous system; IDH, isocitrate dehydrogenase; MGTM, O^6^-methylguanine-DNA methyltransfer; GTR, gross total resection;

## DISCUSSION

Recently, the revised World Health Organization Classification of Tumors of the Central Nervous System has published on Acta Neuropathologica [1]. It brought several new perspectives towards traditional pathology: promote the development of pathology into a molecular era; acknowledge the existence tumor heterogeneity, especially in the biological phenotype; emphasize the role of molecular pathology in personalized treatment. In the Cox proportional hazard model, we have realized the robust potential of the new classification scheme in predicting prognosis. Since the 2016 classification system of gliomas has integrated with 1p/19q and IDH status, the epidemiological characteristics of the new pathology entities also changed. Therefore, this study was designed to uncover the peak age incidence, gender ratio, tumor location and prognosis of the new entities.

In the present study, we found that the incidence and survival of GBM_2016_ has decreased. This situation was directly resulted from the fact that 10 GBMOs with 1p/19q-codeleted and IDH-mutant had been reclassified into grade III according to the 2016 diagnostic criteria. It reduced the total number of WHO grade IV gliomas to some degree. However, GBM was still the most common malignant tumor in the brain, which accounted for 31.0% of all the glioma in our study. GBMO, as its name suggested, was glioblastomas with oligodendroglioma component, which implied decreased malignancy and relatively favorable prognosis [5–8]. Undoubtedly, the deletion of GBMO would attributed to shortened survival of patients with GBM over the next few years.

With one interesting exception, almost all new pathology entities in our study with survival trend followed the principle below: the higher grade, the shorter survival. We noted that patients with IDH-wildtype anaplastic astrocytoma (AA) exhibited worse prognosis than IDH-mutated GBM, which was in accordance with the results reported by Hartmann et al. [9]. Our data showed the median OS of IDH-wildtype AA was 19.0 months, which was close to the survival of newly diagnosed “classic” GBM [10–12]. With this regard, it seemed to be more rational to group IDH-wildtype AA into WHO grade IV which implied more aggressive treatments were needed in routine clinical course.

In line with previous studies [13, 14], almost all gliomas were more frequent in male than female. In 2015, CBTRUS statistical report of primary brain tumors revealed that approximately 55.0% of the malignant tumors occurred in males (65,056 tumors) and 45.0% in females (51,967 tumors) [14]. This gender ratio was mainly driven by the most common brain malignant tumor—glioma. In the present study, the male-preference was particularly prominent in the cluster of WHO grade III gliomas and IDH-wildtype GBM. A statistical report from West China Glioma Center demonstrated there was a dramatically high incidence of malignant tumor in males [15]. But the mechanisms behind this association were still unclear. Perhaps it was correlated with the phenomenon that male was the major labor force in China and thereby had more exposure chance to harmful substance which signified higher risk for brain neoplasm [16].

In this study, GBM, the most malignant brain tumor, was correlated with the highest median age at diagnosis. GBM was primarily diagnosed at older ages, with higher rates between 75 and 84 years old [17]. Our data showed the median age at diagnosis of IDH-mutant GBM and IDH-wildtype GBM was only 48 years and 50 years, respectively, which was similar with result (51 years) provided by Wang et al. [15]. It's well-grounded that older age was a risk factor and always conferred to shorter survival [18–20]. Therefore, we ascertained that older age was a major reason for the dismal prognosis of patients with GBM.

The majority of gliomas occurred in the frontal and temporal lobes in our dataset which was in accordance with previous studies [14, 15]. We further found that gliomas concurrent with IDH-mutant and 1p/19q-codeleted or with single IDH-mutant were mainly distributed in frontal lobe, while those with IDH-wildtype were prevailed in temporal lobe. It was consistent with our report in 2012 [21].

In the following subgroup analysis, we noted that oligodendroglioma with IDH-mutant and 1p/19q-codeleted was the most common tumor in frontal lobe. Mueller et al. reported the frequency of 1p/19q co-deletion of oligodendroglial tumors in the temporal lobe (23.1%) was significantly less than that in non-temporal lobes (81.7%) [22]. Furthermore, Huang et al., in 2008, found that oligodendroglial tumors located in the non-temporal lobes were significantly more likely to have combined deletion of 1p and 19q compared with tumors arising in the insula, temporal lobe, and temporal with another lobe (*P*=0.001) [23]. It's well-established that oligodendroglioma with 1p/19q-codeleted always had better response to procarbazine, lomustine, and vincristine (PCV) chemotherapy regimen [24–26]. Therefore, in the under-developed areas without resource to detect the 1p/19q status, PCV protocol remained a relatively reasonable alternative for patients with gliomas located in frontal lobe.

Interestingly enough, we noticed primary GBM occupied absolute predominance in temporal lobe which was characterized by low incidence of IDH mutation and dismal prognosis. This finding was consistent with previous research results [21, 27]. While gliomas of insular origin were more likely to be low-grade gliomas, such as IDH-mutant astrocytoma, but were less likely to be glioblastoma and anaplastic astrocytoma. The results implied that gliomas located in different lobes might confer to distinct gene phenotypes, which could provide new insights into potential overlap between different prognostic variables and might help to identify niche locations for glioma cells of origin [28]. But unfortunately, the underlying molecular mechanism of these phenomena still remains unclear.

There are also some limitations in the current study. First, it's a single-institution study. Our department is committed to treating patients with supratentorial lesions and aged from 16 years to 60 years, which will lead to bias, especially in analysis of age at diagnosis. Second, diffuse midline glioma is a new and very important entity in the 2016 WHO Classification of Tumors of the CNS. However, it's not included in this manuscript because of the relatively small sample. Third, the median PFS and OS of several subgroups are not available because of the relatively short follow-up. Therefore, in the future, we will continue our study with long-term follow-up in order to revalidate these results.

In conclusion, the 2016 CNS WHO classification scheme is an independent factor associated with prognosis. This study elucidates the epidemiological characteristics as well as the regional constituents of these new gliomas entities, which could bring insights into tumorigenesis and personalized treatment of Chinese glioma population.

## PATIENTS AND METHODS

### Patients and tumor samples

Records from a consecutive series of 1210 patients with a histological diagnosis of gliomas and complete clinical data from January 2009 to May 2016 were retrospectively enrolled in the present study. All specimens were independently reviewed by 3 experienced neuropathologists (Jumei Wang, Guang Li, and Lin Luo), who were blinded to the clinical outcomes of the patients, according to the 2007 and 2016 CNS WHO criteria [1, 29]. This study was approved by the institutional review board of Capital Medical University. All the participants have provided informed consent for this study.

Tumor location usually refers to the lobe or region of the brain, in which the bulk of the tumor resided. They were specified according to the International Classification of Diseases, version 10 (ICD-10) as previously described by Larjavaara [30]. Besides, we added another location: insular lobe, according to the classification of tumors of limbic and pralimbic systems by Yasargil [31]. The orientation was judged by the MR images, including T1-weighted axial, coronary, and sagital images pre- and post-enhancement and T2-weighted axial images. Progression-free survival (PFS) was designated as the time from the first operation to the time of tumor recurrence. Overall survival (OS) was calculated from the date of surgical resection until death or the last known follow-up. The resection degree was confirmed by postoperative contrast-enhanced MRI within 48-72 hours. All patients enrolled in the present study were treated according to the latest National Comprehensive Cancer Network guideline. The specific treatment protocol has been elaborated detailedly in our previous study [32].

### Evaluation of 1p/19q, IDH and MGMT

1p/19q abnormality was determined by fluorescence in situ hybridization (FISH) with 1p36/1q25 or 19q/19p locus-specific identifier DNA dual color probes (Vysis). The experiment protocol and interpretation principle have been elaborated in a previous study [32]. For each probe, >100 nonoverlapping nuclei were enumerated per hybridization. Tumors with more than 30% of nuclei showing DNA loss were defined as tumor with chromosomal loss. MGMT promoter methylation status was evaluated by methylation specific PCR (MSP) and IDH mutation was detected by sequence analysis, as described in another paper from our team [33].

### Statistical analysis

One-way ANOVA was employed to compare mean age between different groups. Categorical variables were compared using the chi-square test or Fisher exact test, where appropriate. Survival analysis was calculated by the Kaplan–Meier method and group results were compared using the Log-rank test. Multivariate analysis to identify independent prognostic factors was carried out using the Cox proportional hazards model. All statistical analyses were performed with SPSS (version 19.0, Chicago, IL, USA). For all tests, a P value less than 0.05 was considered statistically significant.

## SUPPLEMENTARY MATERIALS FIGURES


