# Supplementary material for: Impact of epidemiological characteristics of supratentorial gliomas in adults brought about by the 2016 world health organization classification of tumors of the central nervous system
Source: Oncotarget. 2016 Nov 24;8(12):20354–61. doi: 10.18632/oncotarget.13555 (PMC5386767; doi:10.18632/oncotarget.13555)
Supplement: Supplementary file 1 [file oncotarget-08-20354-s001.pdf]

Impact of epidemiological characteristics of supratentorial gliomas in adults brought about by the 2016 world health organization classification of tumors of the central nervous system

SUPPLEMENTARY FIGURES

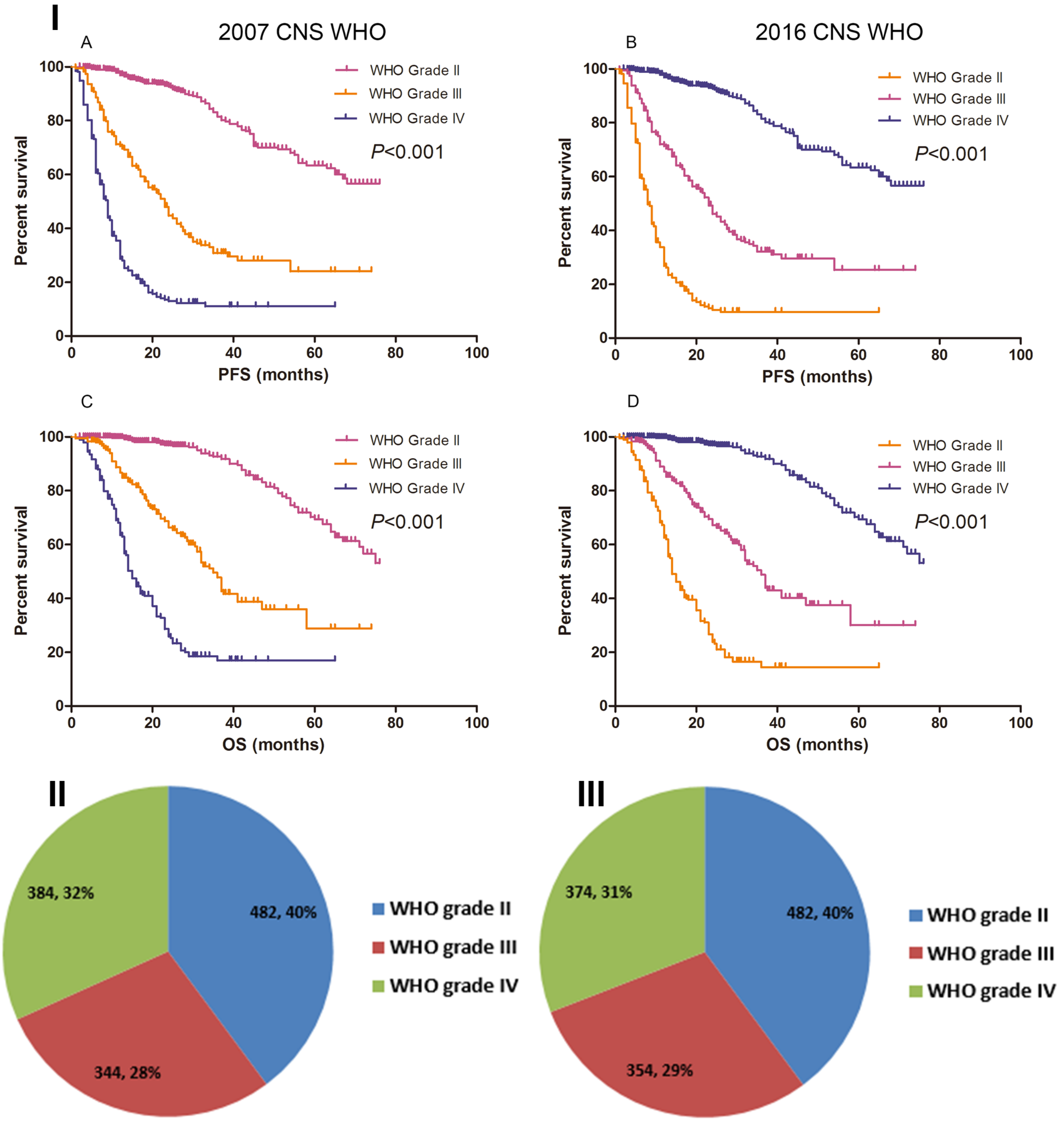

Supplementary Figure S1: The survival rate and specific distribution of different tumor grade in 2007/2016 CNS WHO.

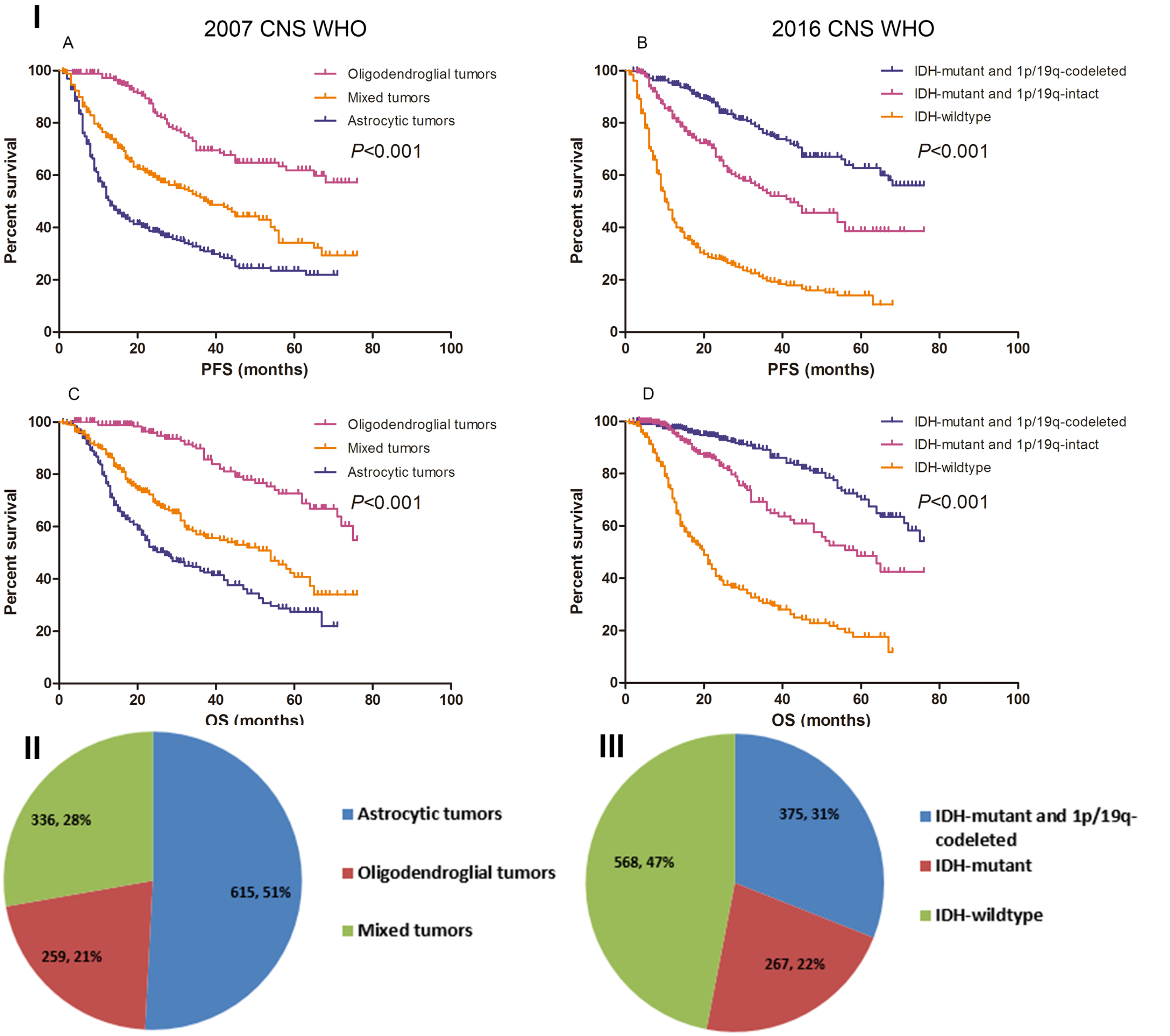

Supplementary Figure S2: The survival rate and specific distribution of different subtypes in 2007/2016 CNS WHO. In the 2007 CNS WHO, astrocytic tumors was the most common glioma in the brain, but conferring the shortest survival time. In the 2016 CNS WHO, gliomas with IDH-wildtype was the most common malignant neoplasm in the brain, conferring the shortest survival time.
